# Long-term outcome following cyclosporine-related neurotoxicity in paediatric allogeneic haematopoietic stem cell transplantation

**DOI:** 10.1038/bmt.2016.232

**Published:** 2016-09-19

**Authors:** K Straathof, P Anoop, Z Allwood, J Silva, O Nikolajeva, R Chiesa, P Veys, P J Amrolia, K Rao

**Affiliations:** 1 Department of Blood and Marrow Transplantation, Great Ormond Street Hospital for children, London, UK

With increasing numbers of children and adults undergoing allogeneic haematopoietic stem cell transplantation (HSCT), neurotoxicity is emerging as an important cause of transplant-related morbidity.^1, 2, 3^ The calcineurin inhibitor cyclosporine A (CI CSA) is the most frequently used agent for prevention of GvHD in both adult and paediatric patients. CSA is an established cause of post-transplantation central nervous system (CNS) toxicity, typically characterised by posterior reversible encephalopathy syndrome (PRES).^4^ Even though neurological complications of CSA seldom result in mortality, the necessitated withdrawal of this potent anti-GvHD agent can have major implications on clinical outcome particularly in the face of on-going GvHD. The spectrum of CSA-related neurological complications has been well characterised in several case series of paediatric HSCT recipients,^5, 6, 7^ however, long-term follow-up data of those children who experience neurotoxicity are lacking. Here, we present the outcome of 26 children who developed CSA-neurotoxicity following allogeneic HSCT.

From a cohort of 569 consecutive paediatric allogeneic HSCT recipients at our institution between 1 January 2003 and 31 December 2013, we retrospectively identified those children who developed neurological complications at any time after the start of conditioning whilst receiving CSA using a computerised search of our patient database. Children with clinical symptoms of PRES or neurological manifestations of thrombotic microangiopathy (TMA)^7^ were included. Those with obvious alternate causes for neurotoxicity were excluded. All children who suffered CNS complications were clinically evaluated by a neurologist. Supplementary investigations such as computed tomography (CT) or magnetic resonance imaging (MRI) scan of head, cerebrospinal fluid analysis and electroencephalogram were used as appropriate to identify the cause. PRES was diagnosed in accordance with its well described MRI appearance.^8^ Hypodense lesions in the parieto-occipital areas were considered to be CT findings consistent with PRES. TMA was diagnosed based on clinical and laboratory features of microangiopathic haemolytic anaemia and thrombocytopenia. GvHD was diagnosed clinically and supported with histological confirmation where possible.^9^ IV CSA administration at a dose of 5 mg/kg/day in two divided doses was commenced either on day −1 (2003–2008) or day −3 (2009 onwards). Levels were monitored twice weekly. Target trough serum levels were 100–150 ng/mL for HLA-identical sibling transplants and 150–200 ng/mL for unrelated donor transplants.

During the 11-year study period, 569 paediatric allogeneic HSCTs were performed at this centre. Twenty-six children developed neurotoxicity associated with CSA (4.6%). Their demographics, disease characteristics and transplant-related parameters are summarised in Table 1. Median age at HSCT was 8.3 years (range 0.5–12.8 years). CSA+MMF was the most commonly employed GvHD prophylaxis (*n*=19), followed by CSA+MTX (*n*=4) and CSA alone (*n*=3). In one patient (subject #19), CSA was changed to Tacrolimus because of renal toxicity before the development of neurotoxicity that is, in this patient neurotoxicity occurred while being treated with Tacrolimus. Twenty-one children experienced CI-related neurotoxicity during their primary transplant procedure and 5 children following their second HSCT, having tolerated CSA without adverse events during their first HSCT.

Median time from HSCT to neurotoxicity was 47.5 days (range day –1 to +545) (Table 1). Clinical symptoms of neurotoxicity and severity are listed in Table 1. Twenty-four out of 26 patients had radiological evaluation of the brain with MRI, CT or both; 19 had classical findings of PRES, 2 showed non-specific changes consistent with encephalopathy and 3 had normal appearances of the brain. CSA level in peripheral blood at the time of presentation with neurotoxicity ranged from 64 to 256 ng/mL (Table 1).

Management of CI-induced neurotoxicity in addition to supportive care consisted of discontinuation of CSA/Tacrolimus in 25/26 patients. Neurological symptoms fully resolved in all patients except in patient #9 who developed TMA and died shortly thereafter and patient #3 who continues to require anticonvulsants despite normalisation MRI appearance. One patient (#12) who presented with seizures on day +24 and had non-specific features on MRI with a normal EEG was continued on IV CSA along with anticonvulsants and had no further neurological events.

After a median of 11 days post onset of CSA neurotoxicity (range 0–597 days) a CI was reintroduced in 18 patients: in 10 patients CSA was used and in 12 patients Tacrolimus, 3 of whom had failed re-challenge with CSA (Table 2). One patient received Tacrolimus after having been successfully re-challenged with CSA (patient #25). Eight of 18 patients re-challenged with a CI had recurrent symptoms of neurotoxicity: 3 following re-challenge with CSA, 2 following re-challenge with Tacrolimus and 3 following re-challenge with CSA as well as Tacrolimus sequentially. In addition to attempted re-challenge with a CI, all but one patient with grade I skin GvHD only before the onset of neurotoxicity, received corticosteroids as GvHD prophylaxis/treatment. Other immunosuppressive agents used are listed in Table 2.

Despite these measures, 7/9 patients (78%) who had no prior GvHD, developed acute/late acute GvHD after onset of CSA neurotoxicity whereas 13/17 had recurrence, persistence or progression of acute/late acute GvHD. Twenty-three patients were evaluable for chronic GvHD as 2 died before day +100 and one patient had second HSCT before day +100. Eighteen out of 23 (78%) had chronic GvHD and this was extensive in 14 patients: 13 patients developed chronic GvHD following CSA-neurotoxicity whereas pre-existing chronic GvHD persisted or progressed in 5 patients (Table 2).

At a median of 176 days (range 7–1889 days) following development of CSA-induced neurotoxicity 15/26 (58%) patients died, most due to progressive GvHD or its complications (Table 2). There were no acute deaths from neurotoxicity except in one patient (subject #9) who had CSA-related TMA on day +61 and died of encephalopathy and respiratory failure on day +68. Currently, 11/26 (42%) of children are alive a median of 8.2 years (range 5.5–13.1 years) after HSCT and a median of 7.8 years (range 5.5–12.1 years) after development of CSA-neurotoxicity. In contrast, during the same study period in our institution, the overall survival at 5 years following allogeneic HSCT was 67.7% for haematological malignancy, 82.1% for immunodeficiency and 87.2% for metabolic disorders. Of the 11 children who survived despite developing CSA-related neurotoxicity, 4 (36%) have a significantly impaired quality-of-life due to sequelae of extensive chronic GvHD.

This case series of paediatric HSCT recipients who developed CSA-related neurotoxicity illustrates that outcome following this complication is notably poor: high non-relapse mortality of 58% and significant morbidity with 36% of survivors living with late effects of extensive chronic GvHD. These findings are similar to those in large case series of adult allogeneic HSCT recipients who developed CSA-related neurotoxicity reporting 43–52% mortality due to progressive GvHD/infection.^10, 11^ In our study, outcome was particularly poor in the 10 patients who had severe GvHD (Grade III/IV) before the development of CSA-neurotoxicity: 8/10 died and 2 are alive with extensive vitiligo. In the same time period at our institution, other patients with severe GvHD, but without the added complication of CSA neurotoxicity had a far superior survival rate of 70%.^12^ Hence it would seem that the development of CSA neurotoxicity and consequent inability to tolerate CI, adversely affected their prognosis. In future studies with larger patient numbers, it would be useful to substantiate our findings with multivariate analyses.

Development of CSA-related neurotoxicity poses a complex clinical management situation as one of the most effective drugs in the treatment and prevention of GvHD needs to be discontinued promptly; sometimes in patients with on-going GvHD. As symptoms of CSA-neurotoxicity usually resolve over several days, re-challenge could be a viable option. Tacrolimus has been used as alternative agent in the event of CSA-related neurotoxicity but as it is also a CI, its use is similarly associated with significant neurotoxicity ^7, 13, 14^ as seen in 5 patients in our case series. Nevertheless, re-challenge with a CI was tolerated in 56% of patients in this series, a similar proportion as that reported by others.^10^ In those patients where re-challenge resulted in recurrence of symptoms and use of CI was precluded permanently, outcome appears particularly dismal with overall survival of 13% (1/8) in this case series.

Evidence for the optimal approach of prophylaxis/management of GvHD when use of CI is contra-indicated is not available. In this case series, all but one patient received corticosteroids. In addition, as the combination of CSA and MMF is the most common GvHD prophylaxis regimen in our centre, the majority of patients were already receiving MMF at the time of development of CSA neurotoxicity. The efficacy of MMF as sole anti-GvHD agent is limited,^13^ and on its own it does not provide satisfactory GvHD prophylaxis or treatment. Sirolimus, which has a completely different mechanism of action to the CI provides another option. Although Sirolimus can also cause neurotoxicity, this has mostly been reported when it has been used in combination with CSA.^15^ In this series, Sirolimus was only used in five patients but this agent may potentially be increasingly used in this clinical situation in future.

In conclusion, this case series illustrates the dismal prognosis in patients following the development of CI-related neurotoxicity and the complexities of managing GvHD in this situation. There is a need for further studies to determine the optimal treatment approach to improve outcome following this rare but serious complication.

## Figures and Tables

**Table 1:** Patient characteristics

*Patient no.*	*Sex/age at HSCT (years)*	*Diagnosis*	*Donor*	*Cell source*	*Conditioning regimen*	*GvHD prophylaxis*	*Day CSA neurotoxicity*	*CSA level at time Neurotoxicity (μg/L)*	*Presentation of neurotoxicity (grading)^a^*	*Description of neurotoxicity/ associated symptoms*
1	M/9	Ph+ ALL	MUD^b^	PBSC	Flu/TBI	CSA/MMF	248	248	PRES (grade 3)	Seizures
2	M/10	MDS	MUD^b^	BM	Flu/Cyclo/Campath	CSA	363	147	PRES (grade 3)	Transient blindness, tremors, severe hypertension, seizures
3	M/4	MDS	MUD	PBSC	Flu/Mel/Campath	CSA/MMF	60	239	PRES (grade 3)	Status epilepticus, tremors
4	F/8	Relapsed ALL	MSD	BM	Cyclo/TBI	CSA	30	256	PRES (grade 4)	Hypertension, reduced consciousness
5	F/8	Ph+ ALL	MSD	BM	Cyclo/TBI	CSA	1	89	PRES (grade 3)	Hypertension, syncopal attack
6	M/5	AML	MMUD	Cord	Bu/Cyclo/Mel	CSA/MMF	16	186	PRES (grade 3)	Hypertension, headaches, seizure
7	M/6	MDS monosomy 7	MSD	BM	Bu/Cyclo/Mel	CSA/MTX	−1	ND^c^	PRES (grade 4)	Seizure
8	M/11	T-NHL	MMUD	PBSC	Cyclo/TBI/Campath	CSA/MMF	115	124	PRES (grade 3)	Seizures, tremors, hypertension
9	M/11	Relapsed AML	MSD	BM	Bu/Cyclo/Mel	CSA/MTX	61	161	TMA (grade 3)	Seizures, hypertension, encephalopathy, cytopenia, renal impairment
10	M/5	JMML-AML	MMUD	Cord	Flu/Treo	CSA/MMF	28	137	PRES (grade 3)	Headache, encephalopathy, seizures, hypertension
11	M/12	AML	MMUD	BM	Bu/Cyclo/Mel/Campath	CSA/MMF	181	201	PRES (grade 4)	Seizure, hypertension, visual impairment
12	M/11	XLP	MMUD	PBSC	Flu/Mel/Campath	CSA/MMF	24	100	PRES (grade 3)	Seizure, hypertension
13	M/7	XLP	MMUD^b^	PBSC	Flu/Mel/Campath	CSA/MMF	202	154	PRES (grade 3)	Seizure, hypertension
14	M/5	HLH	MMUD	Cord	Cyclo/Treo	CSA/MMF	34	106	PRES (grade 4)	Seizures, hypertension
15	F/0.5	HLH	MMUD	Cord	Flu/Treo	CSA/MMF	232	103	PRES (grade 2)	Seizures, hypertension
16	M/2	SCID	MUD	BM	Flu/Mel/Campath	CSA/MMF	77	64	PRES (grade 2)	Seizures, hypertension
17	F/0.5	SCID	MUD	Cord	Flu/Treo	CSA/MMF	15	142	TMA (grade 3)	Headache, hypertension, renal impairment
18	F/7	CID	MMUD	PBSC	Flu/Mel/Campath	CSA/MMF	93	84	PRES (grade 3)	Seizure
19	M/10	CGD	MUD	PBSC	Flu/Treo/Campath	CSA/MMF> TAC/MMF	545	UN	PRES (grade 2)	Seizures, renal impairment
20	F/10	Schwachmann diamond	MUD	PBSC	Flu/Mel/Campath	CSA/MMF	165	109	PRES (grade 3)	Seizures, hypertension
21	F/10	Aplastic anaemia	MSD	BM+cord	Cyclo	CSA/MTX	0	151	PRES (grade 4)	Seizures, hypertension, encephalopathy
22	M/10	Beta thalassaemia major	MSD^b^	PBSC	Mel	CSA/MMF	169	193	PRES (grade 4)	Seizures, encephalopathy
23	F/4	Osteopetrosis	MSD	BM	Bu/Flu	CSA/MMF	16	57	PRES (grade 2)	Seizures
24	F/3	Osteopetrosis	MUD	PBSC	Flu/Treo/Campath/Thiotepa	CSA/MMF	13	175	PRES (grade 4)	Seizures, hypertension, headache
25	F/10	Systemic JIA	MMUD^b^	PBSC	Flu/Mel/Campath	CSA/MMF	35	155	PRES (grade 3)	Seizures, encephalopathy
26	M/8	Adrenoleukodystrophy	MUD	BM	Bu/Cyclo/Campath	CSA/MTX	4	83	PRES (grade 2)	Headaches, seizures

Abbreviations: ALL=acute lymphoblastic leukaemia; AML=acute myeloid leukemia; BM=bone marrow; Bu=busulphan; CGD=chronic granulomatous disease; CID=combined immunodeficiency; CSA=cyclosporine; Cyclo=cyclophosphamide; HLH=haemophagocytic lymphohistiocytosis; JIA=juvenile idiopathic arthritis; MDS=myelodysplastic syndrome; Mel=melphalan; MMF=mycophenolate mofetil; MSD=matched sibling donor; MMUD=mismatched unrelated donor; MUD=mismatched unrelated donor; MTX=methotrexate; ND=not done; T-NHL=T-cell non-Hodgkin lymphoma; Treo=treosulphan; UN=unknown; XLP=X-linked lymphoproliferative disease.

a As per Common Terminology Criteria for Adverse Events.

b Second HSCT.

c This patient developed PRES on the day of starting CSA.

**Table 2:** Immunosuppression, GvHD and outcome following CSA-related neurotoxicity

*Patient no.*	*Immune suppression post CSA toxicity*	*Interval before re-starting CSA/TAC*	*Maximum GvHD pre CSA toxicity acute and chronic*	*Evolution of GvHD post CSA neurotoxicity*		*Outcome/follow-up*^a^ *cause death/current complications*	
				*Acute/late acute*	*Chronic*		
1	TAC^b^, CS, mAb	219	Grade II skin, extensive chronic skin, mouth	Nil	Progressed to extensive skin, mouth and lung	Alive +7.8 years	Extensive bronchiolitis obliterans
2	CS, MMF	NA	Grade I skin	Progressed to Grade II skin	Developed limited skin	Alive +12.1 years	GvHD resolved
3	CSA/TAC^^c^^, CS	20	Grade IV skin and gut	Grade IV skin, gut recurred	Developed extensive skin	Alive +10.3 years	Extensive vitiligo, epilepsy following PRES needing treatment with anti-epileptics
4	Nil	NA	Grade I skin	Grade I skin recurred	Nil	Alive +8.1 years	
5	TAC, CS	132	Nil	Developed Grade III skin, liver	Developed extensive skin, lung	Alive +5.9 years	Bronchiolitis obliterans, bilateral avascular necrosis of the hip
6	TAC, CS, MMF, Siro, mAb, MSC	597	Grade IV skin, gut	Grade IV skin, gut persisted	Developed limited skin, liver	Deceased +1.8 years	Liver GvHD
7	TAC^c^, CS, MMF, mAb	228	Nil	Developed Grade IV skin, gut	Developed extensive skin, gut, liver	Deceased +293 days	Bronchiolitis obliterans/infection
8	CSA/TAC^c^, CS, MMF, MTX, mAb	2	Grade IV skin, gut, extensive chronic skin/gut	Nil	Progressed to extensive skin, gut, liver	Deceased +1.2 years	GvHD, infection (*E. coli* sepsis, pulmonary Aspergillosis)
9	TAC, CS	0	Grade IV skin, gut, liver	Grade IV skin, gut, liver persisted	NE	Deceased +68 days	Infection, TMA
10	CS, MMF	NA	Nil	Developed Grade II gut	Developed limited gut	Deceased +1.6 years	Acute liver necrosis, presumed infection
11	CSA^c^, CS, MMF, Siro	3	Grade II skin	Progressed to Grade III skin	NE^d^	Deceased +275 days	Bone marrow aplasia, fungal chest infection, CMV/adeno/HHV6 viraemia
12	CSA^e^, CS, MMF	10	Nil	Developed Grade II skin	Developed limited skin	Alive +10.4 years	GvHD resolved
13	CS, MMF, mAb	NA	Grade II skin, limited chronic skin	Progressed to Grade IV gut	Limited skin persisted. Developed extensive gut	Alive +7.8 years	GvHD resolved
14	CS, Siro, mAb, MSC	NA	Grade IV skin and gut	Grade IV gut persisted	Developed extensive gut	Alive +6.2 years	GvHD resolved
15	TAC, CS	30	Grade 1 skin	Progressed to Grade IV skin, gut	Developed extensive skin, gut	Deceased +298 days	GvHD, infection (RSV pneumonitis)
16	CSA/TAC^c^, CS, mAb, MSC	4	Grade III skin, gut	Progressed to Grade IV skin, gut, liver	Developed extensive skin, gut, liver	Deceased +253 days	GvHD/infection
17	CSA, CS, MMF, mAb, ATG, MSC	1	Grade IV skin	Grade IV skin persisted Developed Grade IV gut	NE	Deceased +66 days	GvHD, pulmonary haemorrhage
18	CS, MMF, mAb	NA	Grade I gut	Progressed to Grade III skin, gut	Developed extensive skin, gut	Deceased +1.9 years	GvHD
19	TAC^c^, CS, MMF, mAb	0	Grade III skin, gut, liver, extensive chronic skin, gut, liver	Nil	extensive skin, gut, liver persisted	Deceased +1.9 years	Infection (Influenza pneumonitis)
20	CSA^c^, CS, Siro mAb	12	Grade III skin, gut, extensive chronic skin, gut	Grade III skin, gut recurred	Progressed to extensive skin, gut, liver	Deceased +304 days	GvHD, infection (adenoviraemia)
21	CSA^c^, CS	4	Nil	Nil	Nil	Deceased +5.2 years	Pulmonary fibrosis
22	TAC, CS, MMF	343	Grade IV skin, gut	Nil	Developed extensive skin, gut	Alive +10.3 years	Extensive vitiligo
23	CS, MMF	NA	Nil	Developed Grade I skin	Nil	Alive +5.5 years	GvHD resolved
24	CS, MMF, Siro	NA	Nil	Nil	Nil	Deceased +125 days	Pulmonary vasculopathy, infection
25	CSA/TAC, CS, MMF, mAb	5	Nil	Developed Grade IV skin, gut	Developed extensive gut, liver	Deceased +1.6 years	Infection
26	CSA, CS	20	Nil	Developed Grade I skin	Nil	Alive +7.9 years	GvHD resolved

Abbreviations: CS=corticosteroids; CSA=cyclosporine; mAb=monoclonal antibodies including infliximab, daclizumab and/or basiliximab; MMF=mycophenolate mofetil; MSC=mesenchymal stem cells; MTX=methotrexate; NA=not applicable; NE=not evaluable; RSV=respiratory syncytial virus; Siro=sirolimus; TAC=Tacrolimus.

a As of 1 April 2016 except for patient #22 for whom last follow-up was 27 September 2012.

b Tacrolimus started to assess if tolerated in context of potential lung transplantation.

c CSA and/or Tacrolimus restarted but not tolerated.

d NE as received second HSCT.

e CSA continued with clobazam.
